# Activation of Casein Kinase II by Gallic Acid Induces BIK–BAX/BAK-Mediated ER Ca^++^-ROS-Dependent Apoptosis of Human Oral Cancer Cells

**DOI:** 10.3389/fphys.2017.00761

**Published:** 2017-09-29

**Authors:** Meng-Liang Lin, Shih-Shun Chen

**Affiliations:** ^1^Department of Medical Laboratory Science and Biotechnology, China Medical University, Taichung, Taiwan; ^2^Department of Medical Laboratory Science and Biotechnology, Central Taiwan University of Science and Technology, Taichung, Taiwan

**Keywords:** BAX/BAK, BIK, casein kinase II, ER Ca^++^, gallic acid, ROS

## Abstract

Induction of the generation of endoplasmic reticulum (ER) calcium (Ca^++^)-mediated reactive oxygen species (ROS) by gallic acid (GA) has been implicated in the mitochondrial apoptotic death of human oral cancer (OC) cells, but the molecular mechanism by which GA causes ER Ca^++^ release of OC cells to undergo cell death remains unclear. Here, we report that GA-induced phosphorylation of B-cell lymphoma 2 (BCL-2)-interacting killer (BIK) (threonine (Thr) 33/Serine (Ser) 35) and p53 (Ser 15 and Ser 392), Bcl-2-associated x protein (BAX)/BCL-2 antagonist killer 1 (BAK) oligomerization on the ER and mitochondria, rising of cytosolic Ca^+^^+^ and ROS, cytochrome *c* (Cyt *c*) release from the mitochondria, Ψ_m_ loss, and apoptosis were suppressed in cells co-treated with a specific inhibitor of casein kinase II (CK II) (4,5,6,7-tetrabromobenzotriazole). Small interfering RNA (siRNA)-mediated suppression of BIK inhibited GA-induced oligomeric complex of BAX/BAK in the ER and mitochondria, increase of cytosolic Ca^+^^+^ and ROS, and apoptosis, but did not attenuate the increase in the level of Ser 15-phosphated p53 induced by GA. Blockade of p53 expression by short hairpin RNA suppressed BAX/BAK oligomerization and ER Ca^+^^+^–ROS-associated apoptosis induced by GA but did not affect GA-induced phospho-BIK (Thr 33/Ser 35) levels. Induction of mitochondrial Cyt *c* release and ROS generation, increased cytosolic Ca^++^ level, and apoptosis by GA was attenuated by expression of the *BAX* or *BAK* siRNA. Over-expression of BCL-2 (but not BCL-X_L_) inhibited formation of ER oligomeric BAX/BAK by GA. Our results demonstrated that activation of the CK II by GA is required for the BIK-mediated ROS-dependent apoptotic activity of ER-associated BAX/BAK.

## Introduction

Calcium (Ca^++^) is an important second messenger responsible for a variety of the control of cellular processes, including cell growth and survival (Loughery et al., 2014). Transfer of Ca^++^ between the endoplasmic reticulum (ER) and mitochondria confers genotoxic damage-induced apoptosis (Rizzuto and Pozzan, 2006; Kroemer et al., 2007). The initiation of apoptotic process is regulated by the B-cell lymphoma 2 (BCL-2) family of proteins, which can be identified as either pro-apoptotic BCL-2-associated x protein (BAX)/BCL-2 antagonist killer 1 (BAK) or anti-apoptotic BCL-2/BCL-X_L_ proteins (Unger et al., 1993; Cory et al., 2003). In response to apoptotic stimuli, BAX and BAK change their conformations to form oligomers that associate not only with the mitochondrial membrane but also with the ER (Sato and Seiki, 1993; Lu H. L. et al., 2016). ER targeting of oligomeric BAX/BAK causes ER Ca^++^ release, whereas mitochondria-targeted BAX/BAK selectively induces the release of cytochrome *c* (Cyt *c*) from mitochondria (Scorrano et al., 2003; Lu H. L. et al., 2016). The anti-apoptotic function of BCL-2 in the inhibition of BAX-mediated permeabilization of mitochondrial outer membrane was shown to interact with BAX, thereby attenuating the oligomerization and insertion of BAX into the outer mitochondrial membrane (Yin et al., 1994; Cheng et al., 2001; Ding et al., 2010). In addition to the anti-apoptotic function of BCL-2 in the mitochondria, this protein has been reported to modulate ER Ca^++^ homeostasis by the ER targeting (Pinton and Rizzuto, 2006). The protection of ER-targeted BCL-2 against BAX-induced apoptosis has suggested that BCL-2 exerts its anti-apoptotic function to BAX by targeting ER (Wang et al., 2001).

Bcl-2-interacting killer (BIK) is a pro-apoptotic BH3-only member of the BCL-2 family and is found complexed as a heterodimer with BCL-2 or BCL-X_L_ (Elangovan and Chinnadurai, 1997). The phosphorylation at the BIK residues threonine (Thr) 33 and serine (Ser) 35 has been linked to an increase its apoptotic activity (Verma et al., 2001). The kinase responsible for the phosphorylation of BIK Thr 33 and Ser 35 is probably a casein kinase II (CKII)-related enzyme (Verma et al., 2001). The pro-apoptotic activity of BIK is involved in ER-mitochondria Ca^++^ crosstalk by inducing the recruitment and oligomerization of BAX at the ER to confer the stress-induced cell apoptotic death (Mathai et al., 2005). Specific inhibition of *BIK* gene expression by small interfering RNA resulted in aborted p53-induced ER recruitment and oligomerization of BAX, and mitochondrial Cyt *c* release (Mathai et al., 2005).

The mechanisms by which p53 contribute to suppression of tumor growth by mediating apoptosis in response to genotoxic stress have been documented to occur transcription-dependent and transcription-independent pathways (Haupt et al., 2003; Moll et al., 2005). p53 exerts its transcription-independent pro-apoptotic functions through mitochondrial translocation (Moll et al., 2005). Interestingly, p53 lacking transactivation activity can localize to the mitochondrial surface of primary thymocytes undergoing γ-irradiation-induced apoptosis. The formation of the p53–BCL-2/BCL-X_L_ complexes is critical for the induction of permeabilization of the outer mitochondrial membrane by p53 (Mihara et al., 2003), suggesting the physiological relevance of cytoplasmic p53 in regulating the function and integrity of mitochondria *in vivo*. Mitochondrial localization of p53 allows it to induce the release of mitochondrial Cyt *c* by triggering the membrane permeabilization activity of BAX (Mihara et al., 2003; Chipuk et al., 2004). There is convincing evidence that ER-associated p53 can enhance the transfer of Ca^++^ from the ER lumen to the mitochondrial matrix triggering the mitochondrial apoptotic cascade. The findings indicate that apoptotic action of p53 on the ER by interacting with the carboxy-terminal portion of the sarco/ER Ca^++^–ATPase pump enhances Ca^++^ loading resulting in a release of Ca^++^ from ER (Giorgi et al., 2015), indicating that p53 localization to the ER can regulate the response to genotoxic agent-induced apoptosis by modulating the Ca^++^ homeostasis.

The naturally-occurring phenolic compound gallic acid (3,4,5-trihydroxybenzoic acid, GA) exists in the seeds, fruits, and leaves of plants, such as grapes, berries, and tea (Heinonen et al., 1998; Zuo et al., 2002; Shi et al., 2003). It has been demonstrated to possess a variety of pharmacological activities such as antioxidant, anti-inflammatory, antimicrobial, antiviral, and anticancer activities in preclinical studies (Abdelwahed et al., 2007; Kim, 2007; Ozcelik et al., 2011). Experimental evidence supports the fact that GA can selectively induce apoptosis of a variety of human cancer cell lines (Inoue et al., 1995; Elangovan and Chinnadurai, 1997; Yoshioka et al., 2000; Agarwal et al., 2006). The apoptotic action of GA on human cancer cells was attributable to DNA-damage-induced ataxia telangiectasia mutated (ATM) activation (Elangovan and Chinnadurai, 1997; Agarwal et al., 2006), a membrane of the phosphatidylinositol 3-kinase (PI3K)-like family involving in the regulation of cell cycle progression and apoptosis (Guo et al., 2010a,b). We recently showed that GA-induced ER Ca^++^ efflux triggers apoptotic cell death in human oral cancer SSC-4 cells. ER Ca^++^-mediated apoptosis, which occurs due to induction of ER-dependent Ca^++^-mediated ROS generation, leads to activation of mitochondrial apoptotic and ATM-JNK signal pathways (Lu Y. C. et al., 2016). The finding promoted us to further clarify the effect of GA on the induction of ER Ca^++^ release. Toward this end, in this study we investigated the molecular mechanisms associated with GA-induced ER Ca^++^ release.

## Materials and methods

### Cell culture

The human oral cancer SCC-4 cell line was obtained from the Food Industry Research and Development Institute (Hsinchu, Taiwan). The cell line was cultured routinely in Dulbecco's modified Eagle's medium (DMEM) supplemented with 5% fetal bovine serum (FBS) (both from Gibco BRL, Grand Island, NY, USA) and grown in 10-cm tissue culture dish at 37°C in a humidified incubator containing 5% CO_2_.

### Chemicals and reagents

Bismaleimidohexane (BMH), gallic acid (GA), Tris-HCl, and Triton X-100 were obtained from Sigma-Aldrich (St. Louis, MO, USA). GA was dissolved in and diluted with methanol (Daneshfar et al., 2008), and then stored at −20°C as a 100 mM stock solution. Methanol and potassium phosphate were purchased from Merck (Darmstadt, Germany). 4,5,6,7-tetrabromobenzotriazole (TBB) was purchased from Calbiochem (San Diego, CA, USA). Lipofectamine 2000 was obtained from Invitrogen (Carlsbad, CA, USA). FBS, trypsin-EDTA, and glutamine were obtained from Gibco BRL (Grand Island, NY, USA). BAX small interfering RNA (siRNA), BAK siRNA, BIK siRNA, control siRNA, and Western blot luminol reagent were obtained from Santa Cruz Biotechnology (Santa Cruz, CA, USA) (Lin et al., 2014). The BAX siRNA, BAK siRNA, BIK siRNA, and control siRNA were dissolved in RNase-free water.

### Antibodies

Anti-casein kinase II (CK II) antibody was provided by Santa Cruz Biotechnology. Antibodies against BAX, BAK, BCL-2, and BCL-X_L_ were purchased from BD Pharmingen (San Diego, CA, USA). Anti-BIK, phospho (p)-BIK (Thr 33), p-BIK (Ser 35), p-p53 (Ser 15), p-p53 (Ser 392), cytochrome *c* oxidase subunit II (COX2), calnexin, and cytochrome *c* (Cyt *c*) were purchased from Abcam (Cambridge, MA, USA). Antibody against β-actin was obtained from Sigma-Aldrich. Peroxidase-conjugated anti-mouse IgG, -goat IgG, and -rabbit IgG secondary antibodies were purchased from Jackson ImmunoResearch Laboratory (West Grove, PA, USA).

### Plasmid and siRNA transfection

Cells (at 60–70% confluence in a 12-well plate) were transfected with the FLAG-BCL-X_L_ or FLAG-BCL-2 expression plasmid or with BAX siRNA, BAK siRNA, BIK siRNA, or control siRNA using Lipofectamine 2000. The expression of FLAG-BCL-X_L_, FLAG-BCL-2, BAX, BAK, and BIK in transfected cells was assessed by western blotting using antibodies specific to FLAG, BCL-X_L_, BCL-2, BAX, BAK, and BIK.

### Measurement of DNA fragmentation

Histone-associated DNA fragments were determined using the Cell Death Detection enzyme-linked immunosorbent assay (ELISA) kit (Roche Applied Science, Mannheim, Germany). In the vehicle controls, methanol was diluted in culture medium to the same final concentration (0.01%, v/v) as in the medium with GA. Briefly, vehicle- or GA-treated cells were incubated in hypertonic buffer for 30 min at room temperature. After centrifugation, the cell lysates were transferred into an anti-histone-coated microplate to bind histone-associated DNA fragments. Plates were washed after 1.5 h of incubation, and non-specific binding sites were saturated with blocking buffer. Plates were then incubated with peroxidase-conjugated anti-DNA for 1.5 h at room temperature. To determine the amount of retained peroxidase, 2,2′-azino-di-(3-ethylbenzthiazoline-6-sulfonate) was added as a substrate, and a spectrophotometer (Thermo Labsystems Multiskan Spectrum, Frankin, MA, USA) was used to measure the absorbance at 405 nm (Lin et al., 2011).

### Detection of ROS

Briefly, treated cells were then resuspended in 500 μl of 2,7-dichlorodihydrofluorescein diacetate (10 μM) and incubated for 30 min at 37°C. The level of ROS was determined using a FACSCount flow cytometer (Lin et al., 2011; Lu Y. C. et al., 2016).

### Measurement of cytosolic Ca^++^

The Ca^++^ level was determined by measuring the retention of indo-1 acetomethoxy (Indo-1/AM) (Invitrogen, Carlsbad, CA, USA). Briefly, the treated cells were incubated with 3 μg/ml Indo-1/AM for 30 min at 37°C. The cells were then pelleted by centrifugation at 160 × g. The pellets were resuspended and washed twice with PBS. The level of Ca^++^ was evaluated as previously described (Lin et al., 2010).

### Measurement of mitochondrial membrane potential

Mitochondrial membrane potential (ψ_m_) was determined by measuring the retention of the dye 3,3'-dihexyloxacarbocyanine (DiOC_6_). Briefly, treated cells were incubated with 40 nM DiOC_6_ for 30 min at 37°C. Cells were then pelleted by centrifugation at 160 × g. Pellets were resuspended and washed twice with PBS. The Δψ_m_ was determined with a FACSCount flow cytometer (Lin et al., 2011).

### Western blot analysis

Treated or transfected cells were lysed in lysis buffer [50 mM Tris-HCl (pH 8.0), 120 mM NaCl, 1 μg/ml aprotinin, 100 mM Na_3_VO_4_, 50 mM NaF, 0.5% NP-40]. Protein concentration was determined by the Bradford method (Bio-Rad, Hercules, CA, USA). Proteins were separated by electrophoresis on a 10% sodium dodecyl sulfate (SDS) polyacrylamide gel electrophoresis gel and then transferred to polyvinylidene difluoride membranes (Immobilon-P; Millipore, Bedford, MA, USA). Membranes were blocked overnight with phosphate-buffered saline (PBS) containing 3% skim milk and then incubated with primary antibody against CA II, BAX, BAK, BCL-2, BCL-X_L_, BIK, p-BIK (Thr 33), p-BIK (Ser 35), caspase-12, COX2, Cyt *c*, GRP78, p53, or -p-p53 (Ser 15). Proteins were detected with horseradish peroxidase-conjugated goat anti-mouse, goat anti-rabbit, or donkey anti-goat antibodies and Western Blotting Luminol Reagent. To confirm equal protein loading, β-actin was measured (Lu Y. C. et al., 2016).

### Establishment of cell clones permanently expressing p53 shRNA or GFP shRNA

To establish cells stably expressing p53 shRNA or GFP shRNA, cells were transfected using Lipofectamine 2000 with pPuro-p53 shRNA or pPuro-GFP shRNA plasmid. The transfected cells were selected and cloned in the presence of 2 μg/ml puromycin. The efficiency of p53 knockdown was confirmed by western blot analysis with anti-p53 antibody (Lin et al., 2011).

### Subcellular fractionation

Subcellular fractionation was performed according to the protocol of Zong et al. (2003). The treated cells were washed twice with ice-cold PBS and scraped into a 200 mM sucrose solution containing 25 mM HEPES (pH 7.5), 10 mM KCl, 15 mM MgCl_2_, 1 mM EDTA, 1 mM EGTA, and 1 μg/ml aprotinin. The cells were disrupted by passage through a 26-gauge hypodermic needle 30 times and then centrifuged for 10 min in an Eppendorf microcentrifuge (5804R) at 750 × g at 4°C to remove unlysed cells and nuclei. The supernatant was collected and then centrifuged for 20 min at 10,000 × g at 4°C to form a new supernatant and pellet. The resulting pellet was saved as the mitochondrial (Mt) fraction, and the supernatant was further centrifuged at 100,000 × g for 1 h at 4°C. The new supernatant was saved as the cytosolic (Cs) fraction, and the pellet was reserved as the ER/microsomal (Ms) fraction. The resulting Mt and Ms fractions were lysed in RIPA buffer (1% sodium deoxycholate, 0.1% SDS, 1% Triton X-100, 10 mM Tris-HCl [pH 8.0], and 0.14 M NaCl) for Western blot analysis. The purity of each subcellular fraction was confirmed by Western blotting using specific antibodies against the nuclear marker nucleolin, the mitochondrial marker Cox-2, and the ER marker calnexin.

### Statistical analysis of data

Statistical calculations of the data were performed using the unpaired Student's *t*-test and ANOVA analysis. A value of *p* < 0.05 was considered statistically significant.

## Results

### GA induces OC cell apoptosis by inducing the CK II-mediated phosphorylation of BIK

**Figure 1:**
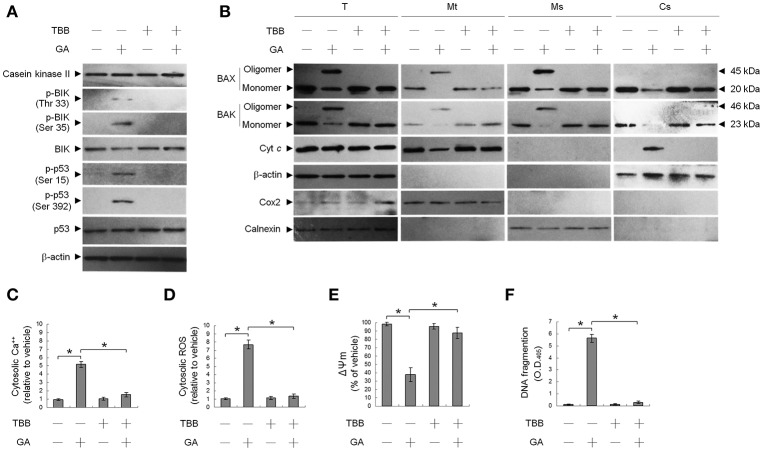
Suppression of gallic acid (GA)-induced BCL-2-associated x protein (BAX)/BCL-2 antagonist killer 1 (BAK) oligomerization in the endoplasmic reticulum (ER), mitochondrial cytochrome c (Cyt c) release, Ψ_m_ loss, and apoptosis by 4,5,6,7-tetrabromobenzotriazole (TBB). **(A,B)** Cells were harvested 36 h after treatment with either vehicle, GA (300 μM), TBB (15 μM), or GA (300 μM) plus TBB (15 μM), and cell pellets were resuspended in hypotonic buffer. Crude homogenates were incubated with 5 mM bismaleimidohexane (BMH) in PBS for 30 min at room temperature and then subjected to subcellular fractionation to obtain the mitochondrial (Mt), ER/microsomal (Ms), and cytosolic (Cs) fractions. In total, 20 μg of total protein from the recovered fractions was analyzed by 10% SDS-PAGE and probed with specific antibodies, as indicated. **(C–F)** Cells were treated with either vehicle, GA (300 μM), TBB (15 μM), or GA (300 μM) plus TBB (15 μM) for for 36 h. The decrease in 3,3′-dihexyloxacarbocyanine fluorescence was measured by flow cytometry. The generation of cytosolic Ca^++^ level and ROS were monitored by measuring increased fluorescence of Indo-1 and 2,7-dichlorodihydrofluorescein by flow cytometry. DNA fragmentation was determined using a Cell Death Detection ELISA kit. The values presented are the mean standard errors from three independent experiments. ^*^Significantly different at *p* < 0.05.

We first investigated the ability of GA to modulate CK II activity, which has been shown to play a key role in targeting of BAX/BAK to ER and the increase of ER Ca^++^ depletion (Verma et al., 2001; Mathai et al., 2005). Western blot analysis revealed that treatment of OC cells with GA resulted in increased in BIK (Thr 33/Ser 35) and p53 (Ser 15 and Ser 392) phosphorylation but had no effect on the expression level of CK II protein (Figure 1A). Activity of CK II appeared to be required for OC cell survival because abolishment of CK II activation by a CK II inhibitor (TBB) causes suppression of cells in the apoptotic induction by GA. Co-treatment of a TBB attenuated GA-induced phosphorylation of BIK (Thr 33/Ser 35) and p53 (Ser 15 and Ser 392), ER and mitochondrial oligomerization of BAX/BAK, increase of ROS, mitochondrial Cyt *c* release, and the alteration of Ψ_m_ (Figures 1A,B,D,E). Increase in cytosolic Ca^++^ level and DNA fragmentation induced by GA was also inhibited in cells co-treatment with TBB (Figures 1C,F). It has been demonstrated that p53 is a physiological substrate of CK II, which is phosphorylated on Ser 392 (corresponding to murine Ser 389) by CK II in response to DNA damage (Meek et al., 1990; Keller and Lu, 2002). These findings suggest that induction of CK II was involved in GA-induced phosphorylation of BIK and p53 and subsequent events of ER–mitochondrial apoptosis in OC cells.

**Figure 2:**
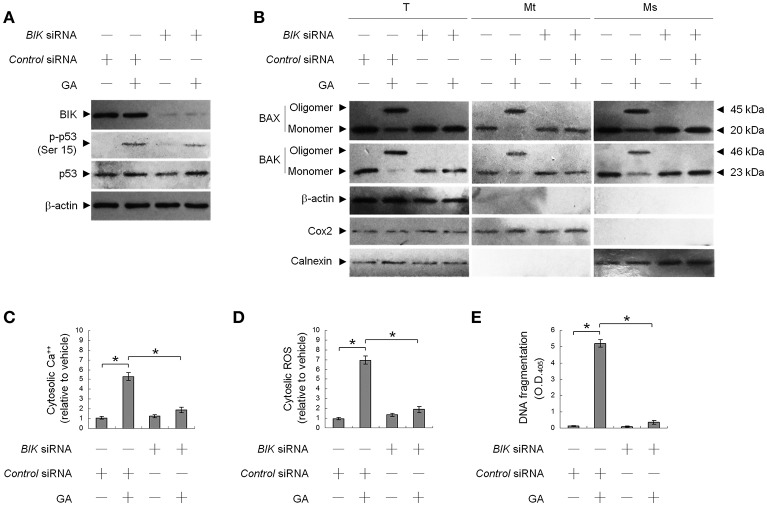
GA-induced activity of Bcl-2-interacting killer (BIK) is responsible for gallic acid (GA)-induced BCL-2-associated x protein (BAX)/BCL-2 antagonist killer 1 (BAK) endoplasmic reticulum (ER) oligomerization, calcium (Ca^++^) efflux from the ER, and cell apoptosis. At 12 h after transfection with control or *BIK* siRNA, the cells were treated with either vehicle or GA (300 μM) for an additional 36 h. **(A,B)** The levels of the indicated proteins in the lysates of the fractions of mitochondrial (Mt), ER/microsomal (Ms), and total cell (T) extracts were determined by Western blot analysis using specific antibodies. Cox-2, calnexin, and β-actin were used as internal controls for the mitochondria, ER, and cytosol, respectively. **(C–E)** The generation of cytosolic Ca^++^ level and ROS were monitored by measuring increased fluorescence of Indo-1 and 2,7-dichlorodihydrofluorescein by flow cytometry. DNA fragmentation was determined using a Cell Death Detection ELISA kit. The values presented are the mean standard errors from three independent experiments. ^*^Significantly different at *p* < 0.05.

**Figure 3:**
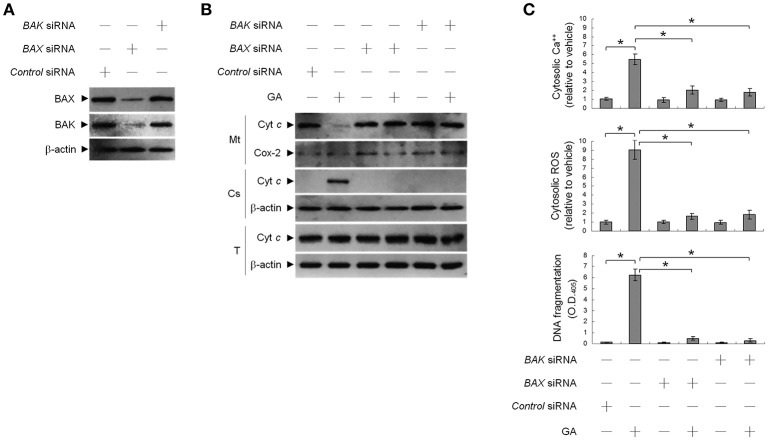
Gallic acid (GA)-induced BCL-2-associated x protein (BAX)/BCL-2 antagonist killer 1 (BAK) apoptotic activity modulates the release of endoplasmic reticulum (ER)-associated calcium (Ca^++^), mitochondrial Cyt c release, and apoptosis. At 12 h after transfection with control, *BAX*, or *BAK* siRNA, cells were treated with vehicle or GA (300 μM) for 36 h. **(A,B)** The levels of the indicated proteins in the lysates of the fractions of mitochondrial (Mt) and cytosolic (Cs) and total cell (T) extracts were determined by Western blot analysis using specific antibodies. Cox-2 and β-actin were used as internal controls for the mitochondria and cytosol, respectively. **(C)** The cytosolic levels of Ca^++^, ROS, and DNA fragmentation were determined by measuring increased Indo-1 fluorescence and 2,7-dichlorodihydrofluorescein using flow cytometry and a Cell Death Detection ELISA kit, respectively. The values presented are the mean standard errors from three independent experiments. ^*^Significantly different at *p* < 0.05.

This raised an interesting possibility that BIK may be a critical regulator of the ER targeting of BAX/BAK by GA in the OC cells. To confirm the role of BIK in BAX/BAK-mediated ER Ca^++^ homeostasis, we employed siRNA to knockdown BIK. siRNA-mediated targeting of *BIK* inhibited induction of BAX/BAK oligomerization in the ER, cytosolic Ca^++^, and ROS elevation, and DNA fragmentation by GA, but there was no effect on the level of Ser-15-phosphated p53 (Figure 2). To address whether GA-induced BAX/BAK apoptotic function linked the induction of ER Ca^++^ release and mitochondrial death signal, cells were transfected with siRNA targeting *BAX* or *BAK*. Immunoblot analysis confirmed the specific knockdown of the expression of BAX or BAK (Figure 3A). Figures 3B,C show that silencing of *BAX* or *BAK* expression by siRNA blocked GA-induced elevation of the cytosolic Ca^++^ concentration, ROS production, release of Cyt *c* from mitochondria, and DNA fragmentation compared to cells transfected with control siRNA. These results indicate that GA-induced ER-associated apoptosis was dependent on the pro-apoptotic activity of CK II–BIK-mediated ER oligomeric BAX/BAK.

### Ser 15 phosphorylation of mutant p53 (P151S) protein involves in BAX/BAK-mediated apoptosis caused by GA

**Figure 4:**
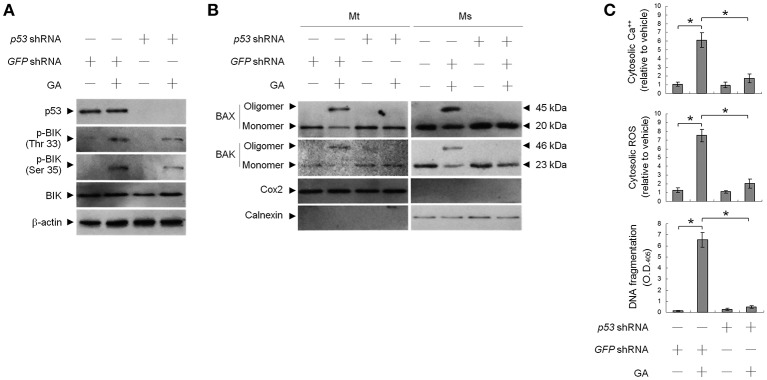
Requirement of phospho-p53 (Ser 15) for targeting of BCL-2-associated x protein (BAX)/BCL-2 antagonist killer 1 (BAK) to the endoplasmic reticulum (ER) by gallic acid (GA). p53 shRNA cells were treated with vehicle or GA (300 μM) for 36 h. **(A,B)** The levels of the indicated proteins in the lysates of the fractions of mitochondrial (Mt) and ER/microsomal (Ms) extracts were determined by Western blot analysis using specific antibodies. Cox-2 and calnexin were used as internal controls for the mitochondria and ER, respectively. **(C)** The generation of cytosolic Ca^++^ level and ROS were monitored by measuring increased fluorescence of Indo-1 and 2,7-dichlorodihydrofluorescein by flow cytometry. DNA fragmentation was determined by using a Cell Death Detection ELISA kit. The values presented are the mean standard errors from three independent experiments. ^*^Significantly different at *p* < 0.05.

Previous work has shown that OC SCC-4 cells harbor p53 mutation (codon 151 proline to serine) (Kim et al., 1993). To ask whether mutant p53 modulated GA-induced BAX/BAK-mediated apoptotic death, we used cells stably expressing a shRNA to knock down p53 and examined the effect of GA on apoptosis induction. p53 protein level was reduced in cells expressing the p53 shRNA, demonstrating efficient and stable knockdown (Figure 4A). No change in DNA fragmentation was observed in vehicle-treated p53 shRNA–transfected cells compared to vehicle-treated non-specific GFP shRNA control cells. Expression of p53 shRNA in cells resulted in attenuation of GA-induced cytosolic Ca^++^ increase, BAX/BAK oligomer formation into the ER and mitochondria, and DNA fragmentation (Figures 4B,C). However, p53 shRNA expression had no detectable effect on the level of BIK Thr 33/Ser 35 phosphorylation (Figure 4A). These data indicate that Ser 15 phosphorylated mutant p53 (P151S) participates in the activation of GA-induced ER oligomeric BAX/BAK-mediated apoptosis.

### Deregulated BCL-2 and BCL-X_L_ involved in GA-induced oligomerization of BAX/BAK at the ER and apoptosis

**Figure 5:**
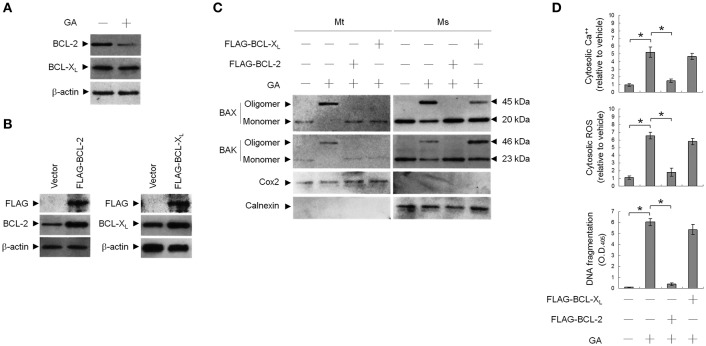
Involvement of decreased B-cell lymphoma 2 (BCL-2) protein level in GA-induced BCL-2-associated x protein (BAX)/BCL-2 antagonist killer 1 (BAK) oligomerization in the endoplasmic reticulum (ER). At 12 h after transfection with vector alone, FLAG-BCL-2, or FLAG-BCL-X_L_, cells were treated with vehicle or GA (300 μM) for 36 h. **(A,B)** Expression levels of BCL-2 and BCL-X_L_ in lysates prepared from cells treated with GA or transfected with vector alone, FLAG-BCL-2, or FLAG-BCL-X_L_. FLAG-BCL-2, FLAG-BCL-X_L_, BCL-2, and BCL-X_L_ were detected with the antibodies shown. **(C)** The levels of the indicated proteins in the lysates of the fractions of mitochondrial (Mt) and ER/microsomal (Ms) and total cell (T) extracts were determined by Western blot analysis using specific antibodies. Cox-2 and calnexin were used as internal controls for the mitochondria and ER, respectively. **(D)** The generation of cytosolic Ca^++^ level and ROS were monitored by measuring increased fluorescence of Indo-1 and 2,7-dichlorodihydrofluorescein by flow cytometry. DNA fragmentation was determined by using a Cell Death Detection ELISA kit. The values presented are the mean standard errors from three independent experiments. ^*^Significantly different at *p* < 0.05.

To address whether induction of BAX/BAK ER targeting and apoptosis by GA was associated with decreased BCL-2 protein levels (Figure 5A), transient ectopic FLAG-tagged BCL-2 or BCL-X_L_ was expressed in cells. Expression levels of BCL-2 and BCL-X_L_ were confirmed by western blotting using FLAG-, BAX-, and BAK-specific antibodies (Figure 5B). Ectopic expression of BCL-2, similar to that of BCL-X_L_, suppressed BAX/BAK oligomerization in the mitochondria. In contrast to BCL-2, ectopic expression of BCL-X_L_ did not completely inhibit the increase in cytosolic Ca^++^, ROS, and DNA fragmentation with GA (Figure 5D). BCL-2 (but not BCL-X_L_) overexpression attenuated the GA-induced ER localization and oligomerization of BAX/ BAK (Figure 5C). These results demonstrate that a decrease in the deregulation of BCL-2 is associated with GA-induced apoptotic potency of oligomeric BAX/BAK in the ER of OC cells.

## Discussion

Based on the present observations and data from our previous studies (Lu Y. C. et al., 2016) indicate that CK II-mediated Thr 33/Ser 35-phosphorylated forms of BIK appears to serve a modulator in initiating the ER Ca^++^-mediated production of ROS through a oligomeric BAX/BAK-regulated mechanism in GA-treated OC cells. In view of observed suppression of GA-induced BIK (The 33/Ser 35) phosphorylation, BAX/BAK ER oligomerization, ER Ca^++^ and mitochondrial Cyt *c* release, ROS generation, and apoptosis by co-treatment with an ATP/GTP competitive inhibitor of CK II inhibitor (TBB), it is logical to suggest that CK II activity has physiological relevance related to modulating survival of OC cells *via* the regulation of BIK–BAX/BAK-dependent ER pathway. Characterization of CK II as an *in vivo* target molecule for GA does not rule possible involvement of protein kinase B (Akt) in the process, as evidence exists that BAX and BAK change their conformation conformations to form oligomers at the ER required AKT inactivation by reducing in its phosphorylation at Ser 473 (Lin et al., 2014). Although the Akt hyper-activation can be promoted by the induction of the phosphorylation of Akt Ser 129 with CK II to contribute anti-apoptotic function of Akt (Di Maira et al., 2005; Ruzzene et al., 2017). This observation, however, is used a phosphatase and tensin homolog deleted on chromosome 10 (*PTEN*)-null human leukemia Jurkat T cells for functional assays (Ruzzene et al., 2017). The finding that Akt was found to be constitutively upregulated in PTEN-deficient human leukemia Jurkat T cells (Di Maira et al., 2005). Further studies are required to better understand the coordinated effect of CK II and Akt on the ER BAX/BAK-mediated apoptosis caused by GA in wild-type PTEN-carrying OC SCC-4 cells (Kubo et al., 1999).

Ser phosphorylation is implicated in stimulating transcriptional activation of p53-targeted genes (*p21, BAX*, and *BAK*) (Loughery et al., 2014) and adopting in a wild-type conformation of p53 (Ullrich et al., 1993). This phosphorylation also contributes to the pro-apoptotic function of p53 in the DNA-damage response (Meek, 2009). In the present study, we have used the SSC-4 cells, which possess a missense mutation in codon 151 of exone 5 (C → T transition) resulting in generation of mutant p53 (P151L) (Kim et al., 1993) and loss of p53 transcriptional activity (Xie et al., 2013). The function of mutant p53 (P151L) has been studied and found to exhibit oncogenic activity in orthotopic xenograft nude mouse (Sano et al., 2011). Consistency, our data indicate that p53 (P151L) lose its transcriptional activity for targeted genes, as evidence fails to induce an increase in the level of p21, BAX, and BAK proteins after treatment with GA; although the treatment induced ER oligomeric BAX/BAK-mediated apoptosis. Despite the fact that the result of p53 (P151L) gain-of-function in the promotion of tumor progression in SCC cell lines (Xie et al., 2013), loss of p53 (P151L) expression by shRNA sensitizes diverse SCC cells to anoikis induction. The oligomerization of BAX and BAK in the ER and apoptosis of SCC-4 cells induced by GA was attenuated by p53 shRNA. A structure-function analysis of p53 mutant proteins reveals that p53 transactivation domain mutants still had some suppression activity (Unger et al., 1993). It is known that BCL-2 can specifically inhibit p53-dependent apoptosis (Hemann and Lowe, 2006). The present study found a decrease in BCL-2 level in GA-treated SSC-4 cells. Using ectopically expressed FLAG-BCl-2 or FLAG-BCL-X_L_, it was found that the GA-induced oligomerization of BAX/BAK in the ER was suppressed by BCL-2. Evidently, these results raised the possibility that GA-induced Ser 15- and 392-phosphorylated forms of p53 (P151L) can act in a negative regulatory effect to control BCL-2 expression and modulates the recruitment of oligomeric BAX and BAK to the ER, although mutant form of p53 (P151L) have lost their transactivation function. In summary, our data provide exciting new insights into therapeutic activity and anti-OC mechanism of GA.

## Author contributions

SC and ML developed the concept of the study, designed the experiments, and wrote the manuscript. ML performed the experiments, collected the data, and performed statistical analysis. SC interpreted the data, supervised this work, and critically revised the manuscript. All authors have read and approved the final manuscript.

### Conflict of interest statement

The authors declare that the research was conducted in the absence of any commercial or financial relationships that could be construed as a potential conflict of interest.
